# Associations Between Psychosocial Well-Being, Stressful Life Events and Emotion-Driven Impulsiveness in European Adolescents

**DOI:** 10.1007/s10964-021-01533-w

**Published:** 2021-11-09

**Authors:** Stefanie Do, Juul M. J. Coumans, Claudia Börnhorst, Hermann Pohlabeln, Lucia A. Reisch, Unna N. Danner, Paola Russo, Toomas Veidebaum, Michael Tornaritis, Dénes Molnár, Monica Hunsberger, Stefaan De Henauw, Luis A. Moreno, Wolfgang Ahrens, Antje Hebestreit

**Affiliations:** 1 Leibniz Institute for Prevention Research and Epidemiology, Achterstrasse 30, 28359 Bremen, Germany; 2 Faculty of Mathematics and Computer Science, University of Bremen, Bibliothekstrasse 5, 28359 Bremen, Germany; 3 Department of Health Psychology, Faculty of Psychology, Open University of the Netherlands, Valkenburgerweg 177, 6419 AT Heerlen, The Netherlands; 4 Copenhagen Business School, Dalgas Have 15, 2000 Frederiksberg, Denmark; 5 Altrecht Eating Disorders Rintveld, Wenshoek 4, 3705 WE Zeist, The Netherlands; 6 Department of Clinical Psychology, Utrecht University, Utrecht, The Netherlands; 7 Institute of Food Sciences, National Research Council, Via Roma 64, 83100 Avellino, Italy; 8 Department of Chronic Diseases, National Institute for Health Development, Hiiu 42, 11619 Tallinn, Estonia; 9 Research and Education Institute of Child health, REF, 138, Limassol Avenue, 2015 Strovolos, Cyprus; 10 Department of Pediatrics, Medical School, University of Pécs, József A. u. 7, 7623 Pécs, Hungary; 11 University of Gothenburg, School of Public Health and Community Medicine, Institute of Medicine, Box 453, SE-405 30, Gothenburg, Sweden; 12 Department of Public Health, Faculty of Medicine and Health Sciences, Ghent University, C. Heymanslaan 10, ingang 42 - verdieping 5, 9000 Gent, Belgium; 13 GENUD (Growth, Exercise, Nutrition and Development) Research Group, Instituto Agroalimentario de Aragón (IA2), Instituto de Investigación Sanitaria Aragón (IIS Aragón), Centro de Investigación Biomédica en Red Fisiopatología de la Obesidad y Nutrición (CIBERObn), Universidad de Zaragoza (UNIZAR), Facultad de Ciencias de la Salud, Domingo Miral s/n, 50009 Zaragoza, Spain

**Keywords:** Negative urgency, Impulsivity, Mental well-being, Stressful life events, Adolescence

## Abstract

Knowing the extent to which mental well-being and stressful life events during adolescence contribute to personality characteristics related to risk-taking behaviors, such as emotion-driven impulsiveness, is highly relevant for the development of health promotion measures. This study examined whether psychosocial well-being and different stressful life events are associated with emotion-driven impulsiveness. In total, 3,031 adolescents (52% girls; M_age_ = 13.6 years) were included from the I. Family Study, a cross-sectional examination on lifestyle-related behaviors conducted across eight European countries in 2013/14. Linear mixed-effects regression models showed that higher psychosocial well-being was associated with lower emotion-driven impulsiveness independent of socio-demographic, health-related, and parental variables. A higher number of stressful life events was associated with higher emotion-driven impulsiveness. Psychosocial well-being and stressful life events need to be further considered in the development and tailoring of health promotion strategies that aim to reduce emotion-driven impulsiveness.

## Introduction

Adolescence is a critical period in life in which a combination of rapid psychosocial changes, somatic growth, and brain maturation processes provide a fertile ground for developing behavioral patterns maintained throughout the life course (Arain et al., 2013). Investigating associations between mental well-being and emotion-driven impulsiveness in adolescence are needed to develop strategies that improve adolescents’ emotion regulation. Particularly considering stressful life events may provide more nuanced information for tailoring interventions that target emotion-driven impulsiveness. Hence, this study investigates the associations between psychosocial well-being, the presence of stressful life events and emotion-driven impulsiveness in a large European adolescent sample aged 12–18.

During adolescence, heightened responsiveness to incentives and emotionally arousing contexts may influence decision-making and lead to the initiation of risk-taking behaviors that may be detrimental to health (Smith et al., 2013). The dual system model of adolescent brain development suggests two independently developing but interacting brain systems that contribute to risk-taking behaviors in adolescence (Shulman et al., 2016): (1) The socio-emotional system which increases motivation to pursue rewards and peaks in adolescence, and (2) the cognitive control system which controls impulses but develops at a slower pace than the socio-emotional system. The imbalance in developmental pace makes adolescence a life time of heightened emotionality and unstable self-regulatory abilities. This suggests that adolescents are more likely to engage in impulsive actions than individuals of other ages to alleviate negative emotions from aversive states or moods for short-term relief (Hasking & Claes, 2019). Besides, adolescents also differ in their ability to control their impulses which is also dependent on their individual predisposition towards impulsivity. Impulsivity is a multifaceted psychological construct that is associated with maladaptive or problematic behaviors (Whiteside et al., 2005).

One facet of trait impulsivity is emotion-driven impulsiveness, i.e., negative urgency, which is the tendency to act rashly when experiencing negative emotions, such as sadness, anxiety, anger, and distress (Cyders & Smith, 2008). Higher emotion-driven impulsiveness cause individuals to alleviate their negative emotions through different unhealthy behaviors, such as substance abuse (Stautz & Cooper, 2013) or unhealthy eating behaviors (Coumans, Danner, Intemann, et al., 2018). However, research examining factors that could decrease adolescents’ emotion-driven impulsiveness through managing negative emotions more effectively, such as via promoting mental well-being is scarce. A few studies on the association between mental well-being and emotion-driven impulsiveness did not provide findings on psychosocial domains (Ravert & Donnellan, 2020; Rose et al., 2018).

### Psychosocial Well-Being

Negative emotions are associated with lower levels of mental well-being (Diener et al., 2009). Mental well-being can be described as a multidimensional construct with cognitive and affective dimensions comprising of three components: life satisfaction, positive, and negative affect (Diener et al., 2009). There is an overall consensus that mental well-being is a multidimensional concept that subsumes different life aspects and entails the presence of positive factors (Steptoe, 2019). Psychosocial well-being, for example, is one of the dimensions of mental well-being which focusses on psychological (intrapersonal) and social (interpersonal) levels of positive functioning (Burns, 2015), and considers resources like self‐esteem, optimism, and social support (World Health Organization, 2004). Higher psychosocial well-being in youth has been associated with supportive social relationships that in turn may reduce negative emotions through social support (Chen et al., 2017). In this regard, emotion-driven impulsiveness can be understood as lying on the pathway as an underlying trait that subsequently leads to maladaptive behavior (Segerstrom & Smith, 2019). Only few studies examined the association between different mental well-being constructs and emotion-driven impulsiveness (Ravert & Donnellan, 2020; Rose et al., 2018). Furthermore, previous studies have reported that sex, age, country, body mass index (BMI), physical activity, sleep characteristics, parental educational level, and parental impulsive behavior were associated with both mental well-being (Hunsberger et al., 2016; Morris et al., 2007; Thumann et al., 2019) and emotion-driven impulsiveness (Delgado-Rico et al., 2012; Guerrero et al., 2019; Morris et al., 2007). Overall, these results indicate a link between mental well-being and emotion-driven impulsiveness which prompts the necessity to further investigate different mental well-being constructs in order to improve adolescents’ emotion-driven impulsiveness.

### Stressful Life Events

Another dimension of mental well-being is the exposure to stress (Steptoe, 2019). Individuals who report that they are less happy about their life do not only report more negative affect but also experience stress more easily and frequently (Ng et al., 2009). In general, stress is caused by a stressor defined as a perceived threat that may exceed an individual’s capabilities and resources for coping and cause negative emotions, such as distress (Lazarus, 2000). Chronic and repeated exposure to stress can overburden neuroendocrine, autonomic, and immune systems when attempting to maintain homeostasis and, subsequently, harm body and brain functions (McEwen, 2007). This is particularly detrimental in sensitive developmental periods, such as childhood and adolescence (Hughes et al., 2017). Stressful life events in early life have been associated with disturbances in cognitive and socio-emotional development (Pakulak et al., 2018) which play an important role in managing negative emotions. A recent review indicated a lack of knowledge on the salience of different types of stressful life events at different developmental points (Cohen et al., 2019). To date, no research has been conducted on the association between different stressful life events and emotion-driven impulsiveness. As an integral part of psychosocial well-being, stressful life events may provide more nuanced information for tailoring interventions that target emotion-driven impulsiveness.

## Current Study

There is only scarce evidence on the association between mental well-being and emotion-driven impulsiveness. In particular, there is no evidence on psychosocial well-being and stressful life events. To close this gap, these study’s aims were to investigate the associations between psychosocial well-being, the presence of stressful life events, and emotion-driven impulsiveness in a large cross-sectional European adolescent sample aged 12 to 18. Based on current evidence, it was hypothesized that higher levels of psychosocial well-being are associated with lower levels of emotion-driven impulsiveness. The direction of this association remains after adjusting for relevant socio-demographic, health behaviors, and parental emotion-driven impulsiveness. Further, it was hypothesized that a higher number of stressful life events is associated with higher levels of emotion-driven impulsiveness and that the strength of association differs between single stressful life events.

## Methods

### Study Population

In the present study, the analysis sample was drawn from I. Family which is a multicenter study based on the prospective IDEFICS cohort (Ahrens et al., 2017). During the baseline examination (2007–2008) children aged 2 to 9.9 years from Belgium, Cyprus, Estonia, Germany, Hungary, Italy, Spain, and Sweden were enrolled from two communities per country with similar socio-demographic profile. Within each community all children attending kindergartens and primary schools were eligible. Parents were approached via these settings and asked for consent to examine their children. In I. Family (2013–2014), participation was offered again to the IDEFICS children and also to siblings who have not been examined earlier. The cohort study was performed according to the standards of the Declaration of Helsinki. Ethical approval was obtained from the ethics committees by all eight study centers according to local standards. Adolescents and parents provided written consent regarding study participation. Data was collected via physical examinations, biological samples and questionnaires following standardized procedures and instruments (Ahrens et al., 2017).

**Fig. 1:**
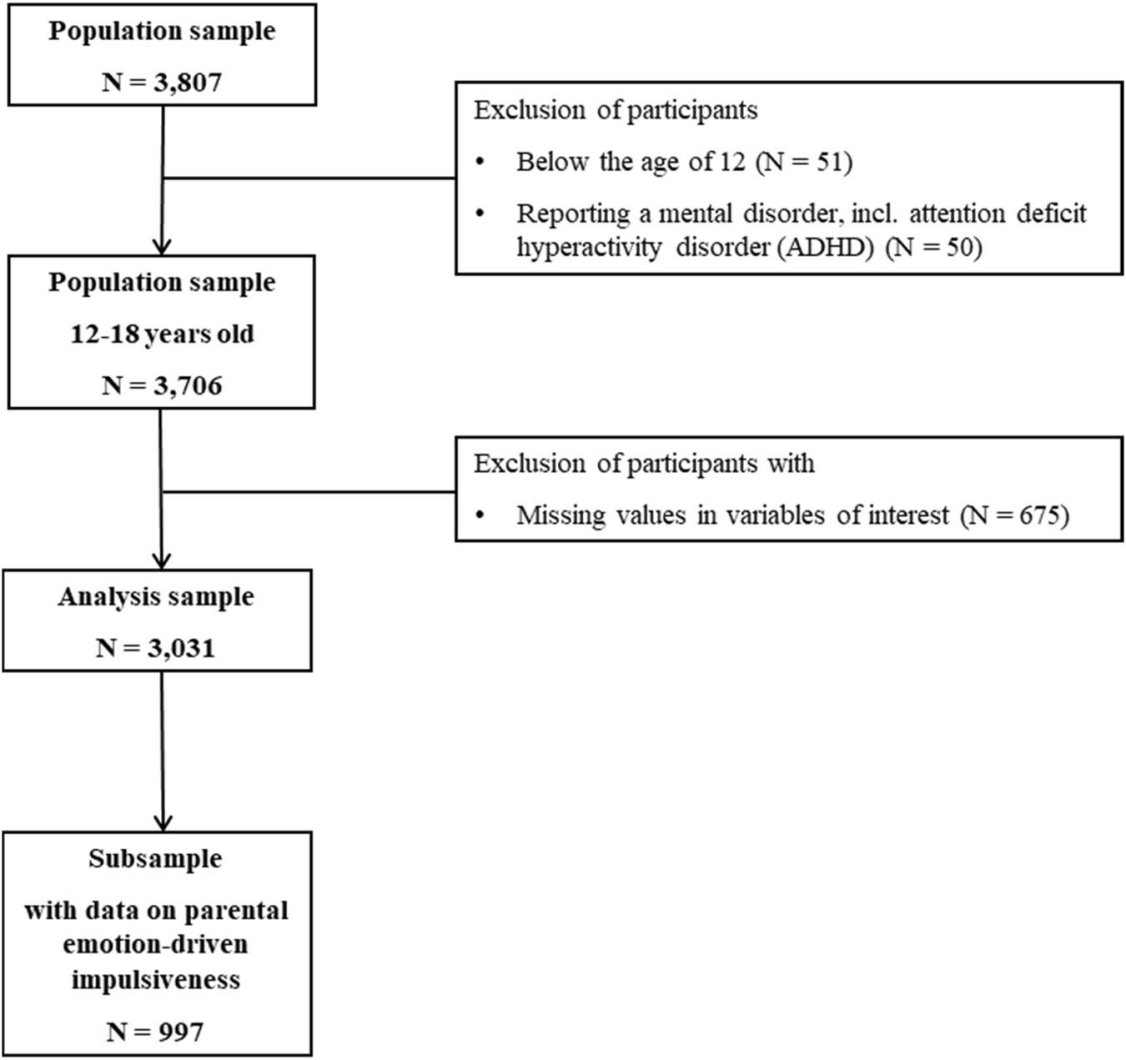
Flow chart of participants

The analysis sample included data from 3,031 adolescents and their siblings between 12 to 18 years of age from 2,732 families. Adolescents with self-reported mental disorders, e.g., attention deficit hyperactivity disorder (ADHD), and with missing information on any study variable were excluded. In a subsample with additional information on parental emotion-driven impulsiveness data from 997 adolescents from 859 families was available. The selection process of the analysis sample and subsample is shown in Fig. 1.

### Emotion-Driven Impulsiveness

The Negative Urgency subscale from the Urgency, Premeditation, Perseverance, Sensation seeking, and Positive urgency (UPPS-P) questionnaire (Whiteside & Lynam, 2001) was used to operationalize emotion-driven impulsiveness. The Negative Urgency subscale included twelve items on a four-point Likert scale ranging from “1” (strongly agree) to “4” (strongly disagree). A sum score was calculated when at least eight items were completed (Whiteside & Lynam, 2001). All items of the original scale, except for one item, were inversely coded to allow all items to run in the same direction. A higher score indicated a higher level of emotion-driven impulsiveness, i.e., being more likely to act rashly in the context of negative emotions; vice versa, a lower score indicated a lower level. The validity and reliability of the UPPS-P have been shown elsewhere (Whiteside & Lynam, 2001; Whiteside et al., 2005). In the present study, the internal consistency was good with a Cronbach’s alpha of 0.88. In all analyses, the negative urgency score for adolescents was used as the outcome variable.

### Psychosocial Well-Being

Psychosocial well-being was measured using 16 items of four subscales of the KINDL^R^ Questionnaire for Measuring Health-Related Quality of Life (HRQoL) in children and adolescents (Ravens-Sieberer & Bullinger, 2000): emotional well-being, self-esteem, family life, and relations to friends. The scale was initially developed in German but was translated to English and other languages. Survey centers were asked to use available language versions. A well-being score was calculated by summing up the 16 items scored on a five-point Likert scale ranging from “0” (Never) to “4” (All the time). Six items of the original scale were inversely coded to allow all items to run in the same direction. Consequently, the well-being score ranged from 0 to 64, with a higher score indicating higher psychosocial well‐being and a lower score indicating lower psychosocial well-being. The validity and reliability of the KINDL^R^ have been shown elsewhere (Bullinger et al., 2008). In the present study, Cronbach’s alpha for this set of items was 0.77, indicating good internal consistency.

### Stressful Life Events

Stressful life events were operationalized as point-in-time adverse life events and assessed by a modified version of the Social Readjustment Rating Scale (Holmes & Rahe, 1967). Respondents were asked to answer the question “Which of the following events did you encounter?” based on a multiple item checklist for lifetime events which, for instance, included parental divorce or separation, death of a family member, parental job loss, long-term separation from a close family member, serious illnesses, or the addition of a new family member. Respondents could tick check boxes to report events. The number of stressful life events in this study ranged from “0” to “5”.

Further, respondents could provide information on their age at event and tick additional check boxes to indicate whether they felt rather strongly or rather little troubled by the event. A recent stressful life event was defined as an event that occurred within the last twelve months by subtracting the age at event from the current age. The timeliness of the event was then categorized as: 1 = no event, 2 = recent event (< = 1 year), and 3 = past event (>1 year). Similarly, the perceived severity of the event was measured as: 1 = no event, 2 = strongly troubled by event, and 3 = little troubled by event.

### Sex

Sex was gauged as male and female (0 = male, 1 = female).

### Age

Age of study participants was measured in years.

### Country of Recruitment

Countries of recruitment included Belgium, Cyprus, Estonia, Germany, Hungary, Italy, Spain, and Sweden (Ahrens et al., 2017).

### Highest Parental Educational Level

Highest parental educational level was determined according to the “International Standard Classification of Education (ISCED)” (UNESCO Institute for Statistics, 2012) (0–2 = low, 3–5 = medium, and 6–8 = high).

### Pubertal Status

Pubertal status was assessed using questions adapted from a self-administered rating scale for pubertal development (Carskadon & Acebo, 1993) and was coded into 0 = no and 1 = yes (yes indicates commencement of menarche for girls and starting or completed voice alterations for boys).

### Body Mass Index (BMI)

BMI was expressed as BMI z-score based on the extended international body mass index cut-offs for thinness, overweight, and obesity (Cole & Lobstein, 2012) to account for differences in BMI by age and sex. BMI z-scores were divided into two categories: overweight/obese (> =25) and thinness/normal weight (<25).

### Sleep Characteristics

Sleep characteristics were determined via self-reported sleep quality derived from the question: “During the past month, how would you rate your sleep quality overall?” (1 = very good, 2 = good, 3 = bad, and 4 = very bad). The two highest response categories “3” (bad) and “4” (very bad) were combined into one category due to their lower frequencies.

### Physical Activity

Physical activity was measured via self-reported sports club membership obtained from one question asking whether the respondents were members of a sports club (0 = no and 1 = yes). Sports club membership was previously found to be significantly associated with meeting the physical activity recommendation (Sprengeler et al., 2017).

### Parental Emotion-Driven Impulsiveness

In certain subgroups with further information on parental emotion-driven impulsiveness, the negative urgency score from biological parents were considered as additional confounding variables.

### Statistical Analysis

Descriptive characteristics are presented as mean, standard deviation, minimum, and maximum for continuous variables and number and percentage for categorical variables. To assess the relationship between the exposure and outcome variables, Spearman’s rank correlation coefficients of emotion-driven impulsiveness, psychosocial well-being as well as stressful life events were first calculated. Second, linear mixed-effects regression analyses were performed to assess the association between psychosocial well-being and emotion-driven impulsiveness. In all analyses, a random effect for family membership was included to account for shared family influences. In the basic model, confounding variables age, sex, country of recruitment, highest educational level of parents, pubertal status, and stressful life events were adjusted for (Model 1a). In the fully adjusted model, BMI, sports club membership, and sleep quality were additionally accounted for (Model 1b). In the subsample analysis, confounding variables from Model 1a and emotion-driven impulsiveness of the biological mother and the biological father were accounted for (Model 1c). Third, associations between the exposure variable stressful life events and the outcome variable emotion-driven impulsiveness were assessed based on the basic model adjusting for age, sex, country, highest educational level of parents, and pubertal status (Model 2). The model with stressful life events was not adjusted for BMI, sports club membership, and sleep quality as these lifestyle variables may lie on the causal pathway. Taking the heterogeneity of stressful life events into consideration, another analysis to assess the associations between the single stressful life events and emotion-driven impulsiveness was conducted (Model 3). Further, sensitivity analyses were conducted for several single stressful life events, which were selected based on their sufficient sample size (at least 15% of the analysis sample): The first sensitivity analysis was performed to assess the associations between the timeliness of single stressful life events and emotion-driven impulsiveness (Model 4a). The second one was conducted to examine the associations between perceived severity of single stressful life events and emotion-driven impulsiveness (Model 4b).

For all regression analyses, predictors and estimated parameters using the maximum likelihood method were included. Unstandardized regression coefficients (B) and 95%-confidence intervals (95%-CI) were reported and the term statistical significance was used to refer to coefficients for which the 95%-CI does not include zero. All analyses were carried out with the statistical software R (Version 4.0.2; (R Core Team, 2020)) using the lme4 package (Bates et al., 2015) for the linear mixed-effects regression.

## Results

### Descriptive Statistics

The analysis sample consisted of 3,031 adolescents aged 12–17.9 years (mean: 13.6 years) with 52% (*N* = 1,587) girls. Main characteristics of the analysis sample are described in Table 1. The majority of adolescents in the sample was thin or normal weight (73%), member of a sports club (58%) and reported a fairly good sleep quality (52%). The most common reported individual stressful life events were the death of a grandparent or other family member (40%), or the addition of new family members (23%) (Supplementary Table 1). Compared to the subsample with additional information on parental emotion-driven impulsiveness, key variables were similarly distributed in both samples with slight differences in stressful life events (27% in the analysis sample reporting at least one stressful life event vs. 24% in the subsample). Further descriptive details on the study population can be found in the supplementary material (Supplementary Tables 2a, 2b, and 2c).

**Table 1:** Descriptive results of the study population

	Analysis sample		Subsample	
	*N* = 3,031		*N* = 997	
	mean (SD)	min–max	mean (SD)	min–max
Negative urgency score	25.2 (7.5)	8–48	25.1 (7.4)	12–48
Well-being score	43.6 (6.6)	9–58	43.8 (6.1)	9–58
Negative urgency score Biological mother	–	–	34.5 (6.5)	12–48
Negative urgency score Biological father	–	–	35.6 (6.8)	12–48
Age	13.6 (1.1)	12–17.9	13.7 (1.2)	12–17.9
	*N* (%)		*N* (%)	
Sex				
female	1,587 (52%)		506 (51%)	
male	1,444 (48%)		491 (49%)	
Highest educational level of parents^a^				
low	175 (6%)		36 (4%)	
medium	1,347 (44%)		409 (41%)	
high	1,509 (50%)		552 (55%)	
Country				
Belgium	64 (2%)		11 (1%)	
Cyprus	744 (25%)		310 (31%)	
Estonia	497 (16%)		111 (11%)	
Germany	243 (8%)		30 (3%)	
Hungary	426 (14%)		208 (21%)	
Italy	552 (18%)		153 (15%)	
Spain	126 (4%)		44 (5%)	
Sweden	379 (13%)		130 (13%)	
Pubertal status				
prepubertal	801 (26%)		257 (26%)	
pubertal	2,230 (74%)		740 (74%)	
BMI^b^				
overweight/obese (> =25)	833 (27%)		265 (26%)	
thinness/normal weight (<25)	2,198 (73%)		732 (74%)	
Physical activity (Sports club membership)				
no	1,261 (42%)		394 (40%)	
yes	1,770 (58%)		603 (60%)	
Sleep quality				
very good	1,116 (37%)		369 (37%)	
fairly good	1,591 (52%)		519 (52%)	
bad/very bad	324 (11%)		109 (11%)	
Stressful life events^b^				
0	1,857 (61%)		707 (71%)	
1	820 (27%)		242 (24%)	
>2	354 (12%)		48 (5%)	

^a^Based on International Standard Classification of Education Maximum (ISCED; maximum of both parents)

^b^Displayed as categorical variables but included as continuous variables in the regression analyses

### Psychosocial Well-Being

With regard to the linear mixed-effects analysis, an inverse association was found between the well-being score and the negative urgency score. This means that for every 5‐point increase in the well‐being score, there was a 0.46 (95%-CI [−0.51, −0.40]) unit decrease in the negative urgency score (Model 1a; Table 2). The association was slightly attenuated in Model 1b when additionally adjusting for BMI, sports club participation, and sleep quality (B = −0.40, 95%-CI [−0.45,−0.34]). Results were also very similar in the subsample analysis with additional adjustment for the negative urgency scores of biological parents in Model 1c (B = −0.16, 95%-CI [−0.19,−0.12]).

**Table 2:** Results from linear mixed-effects regression analyses: Associations between psychosocial well-being, stressful life events (exposures) and emotion-driven impulsiveness (outcome)

	Negative urgency score					
	Analysis sample *N* = 3,031				Subsample *N* = 997	
	Model 1a		Model 1b		Model 1c	
	B	95%-CI	B	95%-CI	B	95%-CI
Well-being score^a^	**−0.46**	**−0.51; −0.40**	**−0.40**	**−0.45; −0.34**	**−0.16**	**−0.19; −0.12**
	Model 2		/	/	/	/
	B	95%-CI				
Stressful life events	**0.08**	**0.05; 0.11**				

Bold letters indicate statistical significance based on confidence limits

All models included a random effect for family affiliation

Model 1a and Model 2: adjusted for age, sex, highest educational level of parents, country, pubertal status, and stressful life events (only in Model 1a)

Model 1b: adjusted as Model 1a plus BMI, sleep quality, and physical activity

Model 1c: adjusted as Model 1a plus negative urgency score for biological mother and father

95%-CI: 95%-confidence interval

^a^1 unit = 5 points

### Stressful Life Events

Moreover, Model 2 revealed a positive association between stressful life events and emotion-driven impulsiveness. This means that for every additional adverse life event, there was a 0.08 (95% CI [0.05, 0.11]) unit increase in the negative urgency score (Model 2; Table 2). The analysis with the single stressful life events displayed the largest positive associations between major frustrations with peers (B = 0.38, 95%-CI [0.31, 0.46]; Table 3) or at school (B = 0.31, 95%-CI [0.25, 0.37]) and the negative urgency score. Respectively, the lowest positive associations were found for addition of a new family member (B = 0.07, 95%-CI [0.02, 0.13]) and moving into a new flat or family home (B = 0.07, 95%-CI [0.02, 0.13]). Additional sensitivity analyses on selected stressful life events, such as addition of new family members and major frustrations at school, indicated that associations with recent stressful life events in comparison to no event were slightly larger than for past stressful life events in comparison to no event (Model 4a; Supplementary Table 4a). For major frustrations at school, associations were also larger when the respondent was strongly troubled by the event (Model 4b; Supplementary Table 4b).

**Table 3:** Results from linear mixed-effects regression analyses: associations between single stressful life events (exposures) and emotion-driven impulsiveness (outcome) in analysis sample

	Negative urgency score	
	analysis sample *N* = 3031	
	Model 3	
	B	95%-CI
*Stressful life events*		
Divorce or separation of parents/step-parents	**0.13**	**0.07; 0.19**
Death of a parent/step-parent	0.10	−0.03; 0.23
Death of a sibling	**0.28**	**0.05; 0.51**
Death of a grandparent or other family member	0.04	−0.01; 0.08
Addition of new family members	**0.07**	**0.02; 0.13**
Job loss of parent/s	**0.10**	**0.02; 0.17**
Major frustrations at school	**0.31**	**0.25; 0.37**
Major frustrations with peers	**0.38**	**0.31; 0.46**
Long-term separation from a close family member	**0.11**	**0.02; 0.20**
Serious diseases, surgery or accidents	**0.15**	**0.08; 0.22**
Serious diseases, surgery or accidents of a family member	0.07	−0.01; 0.14
Moving (into a new flat/family home)	**0.07**	**0.02; 0.13**

Bold letters indicate statistical significance based on confidence limits

All models included a random effect for family affiliation

Model 3: adjusted for age, sex, highest educational level of parents, country, and pubertal status

95%-CI: 95%-confidence interval

## Discussion

Given that mental well-being and stressful life events during adolescence may influence the development of risk-taking behaviors, it is essential to obtain an understanding of their contribution to personality characteristics, such as emotion-driven impulsiveness. Previous studies on the relationship between mental well-being and emotion-driven impulsiveness are scarce and neither provide information on psychosocial domains of mental well-being (Ravert & Donnellan, 2020; Rose et al., 2018) nor close the knowledge gap on the contribution of specific stressful life events at different developmental points (Cohen et al., 2019). As an integral part of psychosocial well-being, investigating stressful life events could provide useful information for tailoring interventions. Therefore, this study aimed to examine the associations between psychosocial well-being, the presence of stressful life events and emotion-driven impulsiveness among adolescents from a large European study conducted in eight countries.

### Psychosocial Well-Being and Emotion-Driven Impulsiveness

Findings in this study show that adolescents with higher psychosocial well-being were less likely to act emotion-driven impulsive after controlling for socio-demographic, health-related, and parental variables. This is in line with previous studies which examined different mental well-being constructs and emotion-driven impulsiveness. The first study demonstrated in a mediation analysis that lower weight-related quality of life was associated with higher emotion-driven impulsiveness through the mediating effects of emotional eating, and food addiction (Rose et al., 2018). Similarly, the second study showed that lower life satisfaction, psychological, and eudemonic well-being was associated with higher emotion-driven impulsiveness, amongst other facets of impulsivity (Ravert & Donnellan, 2020). Generally, different conceptualizations and measures of mental well-being are in place (Lindert et al., 2015); in the present study the concept of psychosocial well-being was closely related to quality of life and subjective well-being (Diener et al., 2009). Hence, findings are consistent with this previous research supporting an inverse relationship between mental well-being and emotion-driven impulsiveness, i.e., higher psychosocial well-being is associated with lower emotion-driven impulsiveness, and vice versa. One explanation is that adolescents with poorer psychosocial well-being tend to have more negative feelings and thoughts about their situation placing a higher load on their working memory, leaving less room for other cognitive control functions for goal-directed behavior (Miyake & Friedman, 2012). Subsequently, they are more likely to engage in impulsive behavior when they experience negative emotions than adolescents who report having better psychosocial well-being; they have more cognitive resources available to focus on long-term goals and tend to engage in more controlled behavior. Another explanation may pertain to individual differences in emotion-driven impulsiveness in which impulsive behavior can improve current mood when feeling negative emotions through immediately rewarding activities, such as alcohol use (Stautz & Cooper, 2013) or snacking behavior (Coumans, Danner, Intemann, et al., 2018). Previous studies illustrating that lower mental well-being is associated with higher emotion-driven impulsiveness, has yet been limited to a small sample of obese adolescents (Rose et al., 2018) or only included adults (Ravert & Donnellan, 2020). Results from this study indicate that psychosocial well-being might be an important predictor for emotion-driven impulsiveness in adolescents as well. These results extend and refine previous research on the relationship between mental well-being and emotion-driven impulsiveness but longitudinal studies are ultimately needed to inform the directionality of this relationship.

### Stressful Life Events and Emotion-Driven Impulsiveness

Furthermore, it is demonstrated that adolescents who reported at least one stressful life event were more likely to act impulsively when experiencing negative emotions after controlling for age, sex, country, highest educational level of parents, and pubertal status. This may be explained by previous research on early life adversity. Early life adversity is known to impair cognitive functions associated with key learning processes, such as emotion regulation (Milojevich et al., 2020; Shonkoff et al., 2011). Additionally, it has been reported that early life adversity may lead to neurocognitive adaptations that cause reduced stress reactivity, a tendency to focus on short-term goals, impulsive response selection, and emotion dysregulation with a preference towards negative states (Lovallo, 2013; Lovallo et al., 2013). Especially children (Lovallo, 2013; Lovallo et al., 2013) are exceptionally vulnerable to the effects of an overactive stress response as they do not have sufficient resources for coping with stressful life events. For instance, results from analyses with single stressful life events illustrate that particularly major frustrations at school and significant frustrations with peers were associated with emotion-driven impulsiveness during adolescence. However, due to the cross-sectional nature of this study, it is also possible that adolescents with higher levels of emotion-driven impulsiveness may experience higher levels of frustrations at school or with peers. Interestingly, results from the sensitivity analyses indicate that more recent stressful life events had a larger effect on emotion-driven impulsiveness. A similar pattern was observed for adolescents who were strongly troubled by stressful life events. It is likely that stressful life events may consume more resources for coping if experienced recently and there are individual differences in perceived severity. Considering the types, timeliness and perceived severity of stressful life events is needed to interpret their association with emotion-driven impulsiveness more precisely.

This study adds to the existing literature on the association between mental well-being and emotion-driven impulsiveness in a large European study of adolescents following a standardized protocol. This unique dataset further facilitated the examination of different types of stressful life events on emotion-driven impulsiveness. However, some limitations must be taken into consideration when interpreting these results. First, the cross-sectional study design neither allowed investigating a temporal relationship nor establishing a causal relationship between the exposure variables and the outcome of interest. In future studies, a longitudinal approach would enable drawing temporal and causal links. Second, we employed data from self-reports which are often sensitive to biases such as social desirability or recall bias. Yet, one of the subscales of the UPPS-P was used to measure the outcome variable emotion-driven impulsiveness, which has been proven to be useful in measuring general behavior over time in a large group of individuals (Cyders & Coskunpinar, 2011). It has also been widely applied to investigate related research questions in the same sample from I. Family (Coumans, Danner, Ahrens, et al., 2018; Coumans, Danner, Intemann, et al., 2018) and in other comparable adolescent samples (Dir et al., 2016; Smith et al., 2021). Third, the medical history of adolescents was self-reported and no more than 50 adolescents have reported a mental disorder. It is likely that adolescents underreported any kind of mental disorders in the analysis sample. Fourth, the operationalization of perceived severity of stressful life events would have been more precise through a weighted score, e.g., events for which adolescents reported to be “rather strongly troubled” could be assigned double the weight as compared to events for which adolescents felt “rather little troubled”. However, these weights would underlie strong assumptions and assignment would be mainly arbitrary. Future studies could assess the perceived severity by investigating the cognitive appraisal of adverse life events or conducting real-time data collection methods, such as ecological momentary assessments. Last, culture may have an influence on mental well-being because of culture-specific patterns in experiencing well-being (Tov & Diener, 2009). However, conclusions related to cultural differences cannot be drawn since there was no data available from representative population samples from each of the countries.

## Conclusion

Due to a life time of heightened emotionality and unstable self-regulatory abilities adolescents are more likely to engage in impulsive actions that are driven by emotions. However, less is known about how to improve adolescents’ impulsive behavior, such as the influence of mental well-being on emotion-driven impulsiveness. In this study, the aim was to investigate the association between psychosocial well-being and stressful life events with emotion-driven impulsiveness in European adolescents aged 12 to 18 years. This study demonstrates that adolescents with higher psychosocial well-being were less likely to act emotion-driven impulsive independent of socio-demographic, health-related, and parental variables. Furthermore, adolescents experiencing a higher number of stressful life events were more inclined to act emotion-driven impulsive. These findings may provide a better understanding of adolescents’ behaviors and an opportunity to tailor interventions that target transdiagnostic factors such as emotion-driven impulsiveness. Considering psychosocial well-being in the development of health promotion strategies might be an important step to improve adolescents’ emotion regulation abilities to avoid engaging in short-term mood alleviation risky behaviors and develop more adaptive coping strategies.

## Supplementary Information

Supplementary Material
