# Supplementary material for: Associations Between Psychosocial Well-Being, Stressful Life Events and Emotion-Driven Impulsiveness in European Adolescents
Source: J Youth Adolesc. 2021 Nov 9;51(6):1106–17. doi: 10.1007/s10964-021-01533-w (PMC9090687; doi:10.1007/s10964-021-01533-w)
Supplement: Supplementary file 1 — Supplementary Material [file 10964_2021_1533_MOESM1_ESM.docx]

**Supplementary Material**

| **Supplementary Table 1** | | |  |
| --- | --- | --- | --- |
| Descriptive results of stressful life events in analysis sample | | |  |
|  | Population sample | Analysis sample | Subsample |
|  | N = 3,807 | N = 3,031 | N = 997 |
|  | **N (%)** | **N (%)** | **N (%)** |
| *Acute stressors* |  |  |  |
| Divorce or separation of parents / step-parents | 617 (16%) | 494 (16%) | 36 (4%) |
| Death of a parent / step-parent | 110 (3%) | 89 (3%) | 6 (0.6%) |
| Death of a sibling | 33 (1%) | 27 (1%) | 5 (0.5%) |
| Death of a grandparent or other family member | 1,442 (38%) | 1,198 (40%) | 410 (41%) |
| Addition of new family members | 865 (23%) | 691 (23%) | 204 (20%) |
| Job loss of parent/s | 352 (9%) | 284 (9%) | 90 (9%) |
| Major frustrations at school | 570 (15%) | 451 (15%) | 155 (16%) |
| Major frustrations with peers | 304 (8%) | 255 (8%) | 91 (9%) |
| Long-term separation from a close family member | 254 (7%) | 209 (7%) | 53 (5%) |
| Serious diseases, surgery or accidents | 363 (10%) | 301 (10%) | 100 (10%) |
| Serious diseases, surgery or accidents of a family member | 422 (11%) | 342 (11%) | 112 (11%) |
| Moving (into a new flat / family home) | 728 (19%) | 587 (19%) | 166 (17%) |
| A respondent can report more than one stressful life event. |  |  |  |

| **Supplementary Table 2a** | | | | |  |  |
| --- | --- | --- | --- | --- | --- | --- |
| Descriptive results of the study population | | | | |  |  |
|  | Population sample | | Analysis sample | | Subsample | |
|  | N = 3,807 | | N = 3,031 | | N = 997 | |
|  | mean (SD) | min-max | mean (SD) | min-max | mean (SD) | min-max |
| Negative urgency score | 25.2 (7.5)^a^ | 8 - 48a | 25.2 (7.5) | 8 - 48 | 25.1 (7.4) | 12 - 48 |
| Well-being score | 43.2 (8.1)^b^ | 0 - 58b | 43.6 (6.6) | 9 - 58 | 43.8 (6.1) | 9 - 58 |
| Negative urgency score Biological mother | 34.5 (6.5)^c^ | 12 - 48^c^ | 34.5 (6.5)^e^ | 12 - 48^e^ | 34.5 (6.5) | 12 - 48 |
| Negative urgency score Biological father | 35.6 (6.9)^d^ | 12 - 48^d^ | 35.6 (6.8)^f^ | 12 - 48^f^ | 35.6 (6.8) | 12 - 48 |
| Age | 13.6 (1.1) | 9.8 - 17.9 | 13.6 (1.1) | 12 - 17.9 | 13.7 (1.2) | 12 - 17.9 |
|  | **N (%)** | | **N (%)** | | **N (%)** | |
| Sex |  | |  | |  | |
| female | 1,931 (51%) | | 1,587 (52%) | | 506 (51%) | |
| male | 1,876 (49%) | | 1,444 (48%) | | 491 (49%) | |
| Highest educational level of parents^g^ |  | |  | |  | |
| low | 226 (6%) | | 175 (6%) | | 36 (4%) | |
| medium | 1,625 (43%) | | 1,347 (44%) | | 409 (41%) | |
| high | 1,812 (47%) | | 1,509 (50%) | | 552 (55%) | |
| missing | 144 (4%) | | - | | - | |
| Country |  | |  | |  | |
| Belgium | 81 (2%) | | 64 (2%) | | 11 (1%) | |
| Cyprus | 988 (26%) | | 744 (25%) | | 310 (31%) | |
| Estonia | 555 (14%) | | 497 (16%) | | 111 (11%) | |
| Germany | 293 (8%) | | 243 (8%) | | 30 (3%) | |
| Hungary | 497 (13%) | | 426 (14%) | | 208 (21%) | |
| Italy | 645 (17%) | | 552 (18%) | | 153 (15%) | |
| Spain | 184 (5%) | | 126 (4%) | | 44 (5%) | |
| Sweden | 564 (15%) | | 379 (13%) | | 130 (13%) | |
| Pubertal status |  | |  | |  | |
| prepubertal | 934 (25%) | | 801 (26%) | | 257 (26%) | |
| pubertal | 2,550 (67%) | | 2,230 (74%) | | 740 (74%) | |
| missing | 323 (8%) | | - | | - | |
| BMI^h^ |  | |  | |  | |
| overweight / obese (>=25) | 1,039 (27%) | | 833 (27%) | | 265 (26%) | |
| thinness / normal weight (<25) | 2,760 (72%) | | 2,198 (73%) | | 732 (74%) | |
| missing | 8 (1%) | | - | | - | |
| Physical activity  (Sports club membership) |  | |  | |  | |
| no | 1,579 (41%) | | 1,261 (42%) | | 394 (40%) | |
| yes | 2,156 (57%) | | 1,770 (58%) | | 603 (60%) | |
| missing | 72 (2%) | | - | | - | |
| Sleep quality |  | |  | |  | |
| very good | 1,405 (37%) | | 1,116 (37%) | | 369 (37%) | |
| fairly good | 1,913 (50%) | | 1,591 (52%) | | 519 (52%) | |
| bad / very bad | 489 (13%) | | 324 (11%) | | 109 (11%) | |
| Stressful life events^h^ |  | |  | |  | |
| 0 | 2,347 (61%) | | 1,857 (61%) | | 707 (71%) | |
| 1 | 1,016 (27%) | | 820 (27%) | | 242 (24%) | |
| >2 | 444 (12%) | | 354 (12%) | | 48 (5%) | |
| ^a^ Based on data available for N = 3,539 | | | | | | |
| ^b^ Based on data available for N = 3,807 | | | | | | |
| ^c^ Based on data available for N = 1,262 | | | | | | |
| ^d^ Based on data available for N = 1,244 | | | | | | |
| ^e^ Based on data available for N = 1,016 | | | | | | |
| ^f^ Based on data available for N = 1,006 | | | | | | |
| ^g^ Based on International Standard Classification of Education Maximum (ISCED; maximum of both parents) | | | | | | |
| ^h^ Displayed as categorical variables but included as continuous variables in the regression analyses | | | | | | |

| **Supplementary Table 2b** | | | | | | |  | |  |  | |  |
| --- | --- | --- | --- | --- | --- | --- | --- | --- | --- | --- | --- | --- |
| Descriptive results of the analysis sample on country level | | | | | | |  | |  |  | |  |
| Country | N (%) | Age (mean) | Sex  (N, %) | | Highest parental educational level (N, %) | | | | Migration background (N, %) | | | |
|  |  |  | female | male | low | medium | | high | no | | yes | |
| Belgium | 64 | 13.2 | 38 (59%) | 26 (41%) | 0 (0%) | 17 (27%) | | 47 (73%) | 62 (97%) | | 2 (3%) | |
| Cyprus | 744 | 13.2 | 375 (50%) | 369 (50%) | 15 (2%) | 305 (41%) | | 424 (57%) | 590 (79%) | | 154 (21%) | |
| Estonia | 497 | 13.8 | 280 (56%) | 217 (44%) | 5 (1%) | 166 (33%) | | 326 (66%) | 467 (94%) | | 30 (6%) | |
| Germany | 243 | 13.5 | 130 (54%) | 113 (46%) | 1 (0%) | 53 (22%) | | 189 (78%) | 206 (85%) | | 37 (15%) | |
| Hungary | 426 | 13.6 | 216 (51%) | 210 (49%) | 15 (4%) | 213 (50%) | | 198 (46%) | 412 (97%) | | 14 (3%) | |
| Italy | 552 | 13.3 | 280 (51%) | 272 (49%) | 106 (19%) | 343 (62%) | | 103 (19%) | 459 (83%) | | 93 (17%) | |
| Spain | 126 | 13.3 | 72 (57%) | 54 (43%) | 1 (1%) | 48 (38%) | | 77 (61%) | 119 (94%) | | 7 (6%) | |
| Sweden | 379 | 13.6 | 196 (52%) | 183 (48%) | 32 (9%) | 202 (53%) | | 145 (38%) | 270 (71%) | | 109 (29%) | |

| **Supplementary Table 2c** | | | | |
| --- | --- | --- | --- | --- |
| Descriptive results of the study population and the excluded participants | | | | |
|  | Analysis sample | | Excluded participants* | |
|  | N = 3,031 | | N = 404 | |
|  | mean (SD) | min-max | mean (SD) | min-max |
| Negative urgency score | 25.2 (7.5) | 8 - 48 | 25.2 (7.9) | 10 - 45 |
| Well-being score | 43.6 (6.6) | 9 - 58 | 44.0 (7.3) | 11 - 58 |
| Age | 13.6 (1.1) | 12 - 17.9 | 13.9 (1.3) | 12 - 17.6 |
|  | **N (%)** | | **N (%)** | |
| Sex |  | |  | |
| female | 1,587 (52%) | | 176 (44%) | |
| male | 1,444 (48%) | | 228 (56%) | |
| Highest educational level of parents^a^ |  | |  | |
| low | 175 (6%) | | 15 (4%) | |
| medium | 1,347 (44%) | | 118 (29%) | |
| high | 1,509 (50%) | | 146 (36%) | |
| missing | - | | 125 (31%) | |
| Country |  | |  | |
| Belgium | 64 (2%) | | 9 (2%) | |
| Cyprus | 744 (25%) | | 111 (27%) | |
| Estonia | 497 (16%) | | 16 (4%) | |
| Germany | 243 (8%) | | 23 (6%) | |
| Hungary | 426 (14%) | | 41 (10%) | |
| Italy | 552 (18%) | | 52 (13%) | |
| Spain | 126 (4%) | | 37 (9%) | |
| Sweden | 379 (13%) | | 115 (29%) | |
| Pubertal status |  | |  | |
| prepubertal | 801 (26%) | | 27 (7%) | |
| pubertal | 2,230 (74%) | | 97 (24%) | |
| missing | - | | 280 (69%) | |
| BMI^b^ |  | |  | |
| overweight / obese (>=25) | 833 (27%) | | 95 (23%) | |
| thinness / normal weight (>=25) | 2,198 (73%) | | 301 (75%) | |
| missing | - | | 8 (2%) | |
| Physical activity  (Sports club membership) |  | |  | |
| no | 1,261 (42%) | | 179 (44%) | |
| yes | 1,770 (58%) | | 221 (55%) | |
| missing | - | | 4 (1%) | |
| Sleep quality |  | |  | |
| very good | 1,116 (37%) | | 164 (40%) | |
| fairly good | 1,591 (52%) | | 193 (48%) | |
| bad / very bad | 324 (11%) | | 47 (12%) | |
| Stressful life events^b^ |  | |  | |
| 0 | 1857 (61%) | | 242 (60%) | |
| 1 | 820 (27%) | | 113 (28%) | |
| >2 | 354 (12%) | | 49 (12%) | |
| * With full information in the outcome variable but missing information in exposure variable and covariates  ^a^ Based on International Standard Classification of Education Maximum (ISCED; maximum of both parents) ^b^ Displayed as categorical variables but included as continuous variables in the regression analyses | | | | |

| **Supplementary Table 3** | | | |
| --- | --- | --- | --- |
| Descriptive results of emotion-driven impulsiveness and psychosocial well-being for different number of stressful life events in analysis sample and subsample | | | |
|  | Stressful life events | Negative urgency score | Well-being score |
|  | N | mean (SD) | mean (SD) |
| Study sample N = 3,031 | 0 | 24.94 (7.56) | 43.98 (6.45) |
|  | 1 | 25.73 (7.43) | 43.20 (6.80) |
|  | >2 | 25.66 (7.21) | 42.58 (6.63) |
| Subsample  N = 997 | 0 | 24.87 (7.45) | 43.85 (6.12) |
|  | 1 | 25.28 (7.35) | 43.76 (6.00) |
|  | >2 | 26.00 (7.02) | 42.54 (5.85) |

| **Supplementary Table 4a** | | |
| --- | --- | --- |
| Results from sensitivity analyses between timeliness of single selected stressful life events (exposures) and emotion-driven impulsiveness (outcome) in analysis sample | | |
|  | Negative urgency score | |
|  | Analysis sample N=3,031 | |
|  | Model 4a | |
|  | B | 95%-CI |
| Death of a grandparent or other family member |  |  |
| no event | reference | |
| past event (>1 year) | 0.03 | -0.02; 0.08 |
| recent event (<=1 year) | 0.05 | -0.03; 0.12 |
| Addition of new family members |  |  |
| no event | reference | |
| past event (>1 year) | **0.06** | **0.01; 0.12** |
| recent event (<=1 year) | **0.14** | **0.02; 0.26** |
| Major frustrations at school |  |  |
| no event | reference | |
| past event (>1 year) | **0.22** | **0.10; 0.33** |
| recent event (<=1 year) | **0.34** | **0.27; 0.41** |
| Moving (into a new flat / family home) |  |  |
| no event | reference | |
| past event (>1 year) | 0.05 | -0.01; 0.11 |
| recent event (<=1 year) | 0.12 | -0.01; 0.23 |
| All models included a random effect for family affiliation. | | |
| Stressful life events were selected based on sufficient sample size (at least 15%). | | |
| Model 4a: adjusted for age, sex, highest educational level of parents, country, pubertal status, and timeliness of single stressful life event | | |
| 95%-CI: 95%-confidence interval | | |
| **Bold letters indicate statistical significance based on confidence limits.** | | |

| **Supplementary Table 4b** |  |  |
| --- | --- | --- |
| Results from sensitivity analyses between perceived severity of single selected stressful life events (exposures) and emotion-driven impulsiveness (outcome) in analysis sample | | |
|  | Negative urgency score | |
|  | Analysis sample N=3,031 | |
|  | Model 4b | |
|  | B | 95%-CI |
| *Selected stressful life event* |  |  |
| Death of a grandparent or other family member |  |  |
| no event | reference | |
| little troubled by event | -0.03 | -0.10; 0.04 |
| strongly troubled by event | **0.08** | **0.03; 0.13** |
| Addition of new family members |  |  |
| no event | reference | |
| little troubled by event | 0.05 | -0.01; 0.11 |
| strongly troubled by event | **0.21** | **0.10; 0.32** |
| Major frustrations at school |  |  |
| no event | reference | |
| little troubled by event | **0.21** | **0.12; 0.30** |
| strongly troubled by event | **0.40** | **0.31; 0.48** |
| Moving (into a new flat / family home) |  |  |
| no event | reference | |
| little troubled by event | 0.03 | -0.04; 0.10 |
| strongly troubled by event | **0.24** | **0.12; 0.37** |
| All models included a random effect for family affiliation. | | |
| Stressful life events were selected based on sufficient sample size (at least 15%). | | |
| Model 4b: adjusted for age, sex, highest educational level of parents, country, pubertal status, and perceived severity of single stressful life event | | |
| 95%-CI: 95%-confidence interval | | |
| **Bold letters indicate statistical significance based on confidence limits.** | | |
